# An immunodominance perspective on a paradoxical phenomenon: discovery and modeling of ragweed and tree sensitization as negative predictors for high mugwort IgE reactivity

**DOI:** 10.3389/falgy.2026.1758315

**Published:** 2026-06-09

**Authors:** Yuqiao Zhang, Xiyuan Yan, Fengxia Yang, Shurong Li, Xueliang Shen, Ruixia Ma

**Affiliations:** 1 The Second Clinical Medical College of Ningxia Medical University, Yinchuan, China; 2 Department of Otorhinolaryngology Head and Neck Surgery, The First People’s Hospital of Yinchuan, Yinchuan, China; 3 Department of Otorhinolaryngology Head and Neck Surgery, Ningxia Medical University General Hospital, Yinchuan, China

**Keywords:** cross-reactivity, machine learning, mugwort allergy, nomogram, predictive model, SHAP, specific IgE, XGBoost

## Abstract

**Background:**

Mugwort allergy poses a significant disease burden in Northwestern China. While sensitization to mugwort can be confirmed through specific IgE testing, the immunological factors that influence the intensity of the IgE response among sensitized individuals remain poorly understood. This study aimed to explore whether sensitization profiles to other common inhalant allergens are associated with the magnitude of mugwort-specific IgE responses in already-sensitized patients, and to characterize the patterns of co-sensitization that may reflect underlying immune mechanisms.

**Methods:**

In this retrospective retrospective study, we enrolled 635 mugwort-sIgE-positive patients from a tertiary hospital. Levels of sIgE to multiple inhalant allergens, including ragweed, trees, and dust mites, were measured. The least absolute shrinkage and selection operator (LASSO) regression identified the most predictive features for high mugwort IgE reactivity (grade ≥4). An XGBoost model was developed and validated on a temporal validation set (*n* = 159). Model interpretability was achieved using SHapley Additive exPlanations (SHAP), and a clinical nomogram was constructed.

**Results:**

Three features—ragweed, tree, and dust mite sIgE concentrations—were selected, all exhibiting negative coefficients. The XGBoost model demonstrated excellent discrimination, with an AUC of 0.851 (95% CI: 0.781–0.919) on the test set and 0.827 on the temporal validation set. SHAP analysis revealed the counterintuitive, negative predictive effects of these allergens on high mugwort reactivity, supporting an immunodominance-based “molecular masking” hypothesis. The derived nomogram and risk stratification system effectively stratified patients, with the high-risk group having an actual positive rate of 85%. Decision curve analysis confirmed the clinical utility of the model.

**Conclusion:**

From an immunodominance perspective, we discovered and modeled sensitization to ragweed, trees, and dust mites as negative predictors for high mugwort reactivity. The interpretable machine learning model provides a precise tool for early identification and stratified management of high-risk mugwort-allergic patients.

## Introduction

The high global prevalence of allergic diseases poses ongoing challenges to their clinical management (1). Mugwort, a predominant airborne pollen allergen in Northwestern China, contributes to a substantial disease burden (2). Current diagnosis of mugwort allergy primarily relies on skin prick testing (SPT) or serum-specific IgE (sIgE) detection. However, sIgE positivity reflects sensitization rather than clinical allergy; many patients remain asymptomatic even with high sIgE levels. The presence and severity of clinical symptoms require comprehensive evaluation based on detailed medical history and clinical assessment. Therefore, relying solely on sIgE levels for reliable pre-symptom warning is challenging, highlighting the need to better understand factors that influence the transition from sensitization to clinical symptoms (3, 4).

A key clinical challenge is the frequent presentation of polysensitization in patients, partly attributable to complex sensitization profiles driven by cross-reactive pan-allergens (e.g., profilins and cross-reactive carbohydrate determinants, CCDs) (5, 6). In recent years, component-resolved diagnostic systems such as ALEX have enabled effective distinction of non-specific sensitization driven by cross-reactive carbohydrate determinants (CCDs) and profilins, thereby providing a more accurate picture of genuine sensitization patterns. However, crude extract-based testing remains widely used in routine clinical practice, and interpreting genuine allergen responses in the context of polysensitization still presents challenges. This complexity limits the efficacy of single-allergen assessment but also underscores the potential of integrating multi-allergen profile information to predict the reaction severity to a primary allergen. Machine learning (ML) techniques have demonstrated unique potential in deciphering such complex, non-linear relationships (7, 8).

Nevertheless, studies leveraging ML to integrate routine multi-sIgE data for predicting the risk of high mugwort reactivity are currently lacking. Therefore, this study aimed to explore whether sensitization profiles to other common inhalant allergens—specifically ragweed, trees, and dust mites—are associated with the magnitude of mugwort-specific IgE responses in patients already sensitized to mugwort. Rather than developing a clinical prediction tool for routine use, we leverage machine learning to characterize the patterns of co-sensitization that may reflect underlying immune mechanisms, with the goal of generating testable hypotheses for future mechanistic and longitudinal studies.

It is important to emphasize that the goal of this study is not to present the machine learning model as a standalone predictive tool independent of biological context, but rather to leverage its capacity to integrate multi-dimensional allergen sensitization data for exploring immunological patterns that may influence the intensity of mugwort sIgE responses. Accordingly, we frame the model findings as a foundation for generating biological hypotheses rather than establishing causal relationships.

## Methods

### Research design and population

**Figure 1:**
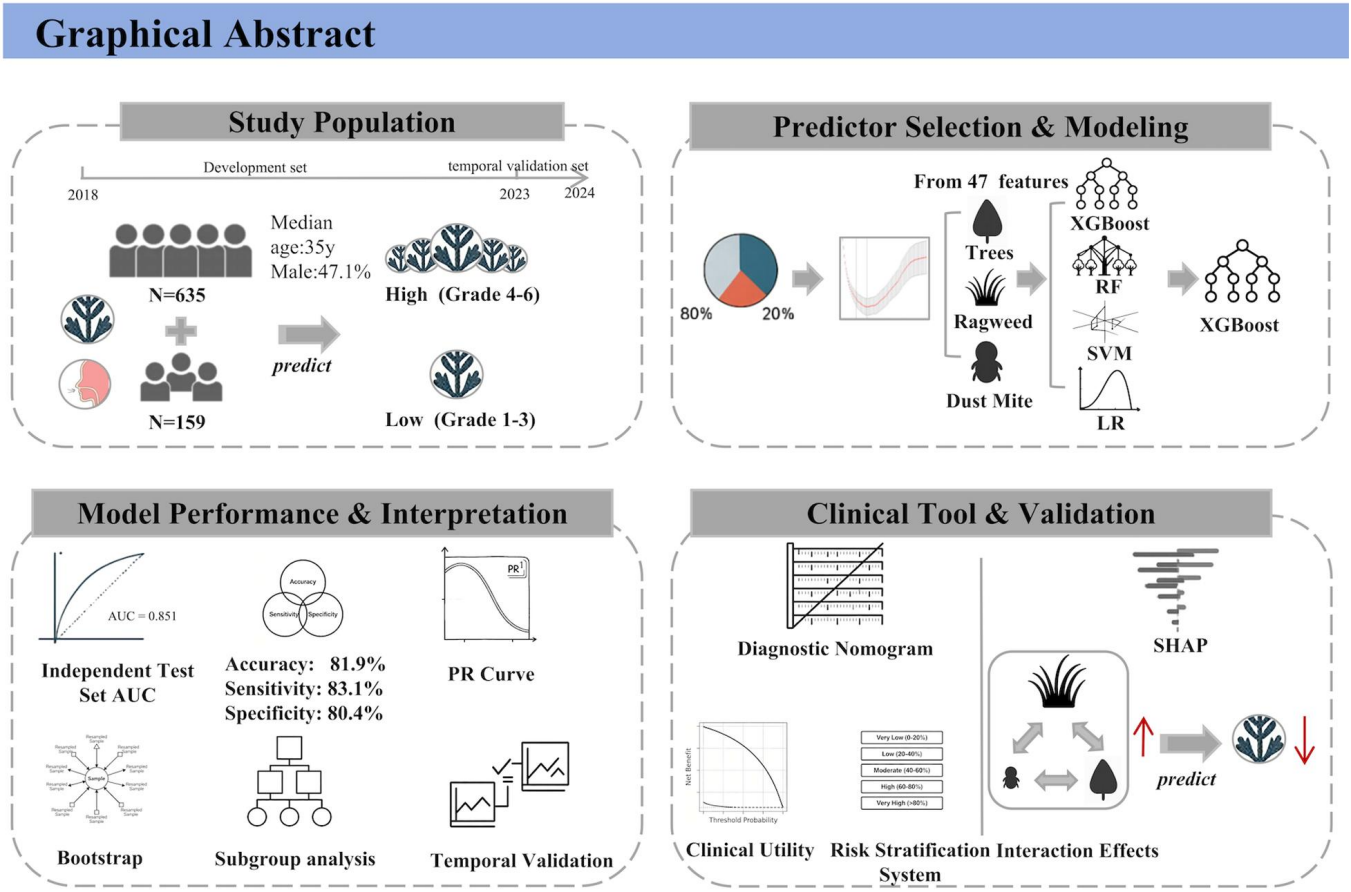
Graphical abstract: An overview of the study design for developing and validating a machine learning model to predict high mugwort IgE reactivity.

This retrospective study aimed to develop and validate a machine learning model for predicting high mugwort IgE reactivity. We consecutively enrolled 635 patients from the Department of Otorhinolaryngology Head and Neck Surgery of the First People's Hospital of Yinchuan between 2018 and 2023, who tested positive for mugwort sIgE, constituting the model development set. An additional 159 patients enrolled from January to December 2024 served as a temporal validation set. An overview of the study design is presented in Figure 1.

The inclusion criteria were: (1) patients during the symptomatic phase of AR, presenting with paroxysmal sneezing, watery rhinorrhea, nasal itching, and congestion, along with signs of pale and swollen nasal mucosa, edematous inferior turbinate, and abundant watery secretions, all meeting the diagnostic criteria of the Chinese Guidelines for the Diagnosis and Treatment of Allergic Rhinitis; (2) availability of complete clinical-demographic information and allergen test results, with mugwort sIgE ≥0.35 U/mL (9).

In our institutional clinical practice, skin prick testing (SPT) and serum sIgE detection are typically used as alternative diagnostic approaches rather than being performed concurrently for all patients. The patients included in this study were selected based on serum sIgE results, which represent the first-line diagnostic method for allergic rhinitis at our center, with its diagnostic value recognized by the Chinese Guidelines for the Diagnosis and Treatment of Allergic Rhinitis. All included patients were in the symptomatic phase and met the clinical diagnostic criteria of the guidelines; therefore, sIgE positivity combined with typical clinical symptoms is sufficient to support the diagnosis of allergic rhinitis.

Exclusion criteria were: (1) patients with immune system diseases or receiving immunosuppressive therapy; (2) patients with malignant tumors or severe chronic diseases; (3) pregnant women; (4) patients who had used systemic corticosteroids within the past three months.

The study protocol was approved by the Ethics Review Committee of the First People's Hospital of Yinchuan (Approval No: 2023-028), and informed consent was obtained from all participants.

Patients were consecutively enrolled between 2018 and 2023, covering all seasons throughout the year. In the Ningxia region, the pollen seasons for mugwort and ragweed are mainly concentrated in July and August (2); therefore, while sample collection has seasonal characteristics, the year-round enrollment design ensures the generalizability of the study findings.

### Data collection and preprocessing

Specific IgE antibodies were measured using the ImmunoCAP® system (Phadia AB, Sweden), a quantitative fluorescence enzyme immunoassay. The sensitivity and specificity of this method are ISO 15189-certified, enabling accurate detection of IgE antibody levels against specific allergens in serum. Ten inhalant allergens were tested: tree mix (poplar/willow/elm), common ragweed pollen, mugwort pollen, house dust mite mix (*Dermatophagoides pteronyssinus/Dermatophagoides farinae*), hay dust, cat epithelium, dog epithelium, German cockroach, mold mix (*Penicillium notatum/Cladosporium herbarium/Aspergillus fumigatus/Alternaria alternata*), and hops. Eleven food allergens were also tested: egg, milk, peanut, soybean, beef, mutton, sea fish mix (cod/lobster/scallop), shrimp, crab, fruit mix (peach/apple/mango/lychee/strawberry), and nut mix (cashew/pistachio/hazelnut/almond/walnut). According to the Chinese Guidelines for the Diagnosis and Treatment of Allergic Rhinitis (9), a specific IgE concentration ≥0.35 U/mL (corresponding to Grade ≥1) was defined as a positive result. Test results were classified into Grades 0–6 based on concentration ranges as follows: Grade 0: <0.35 U/mL; Grade 1: 0.35–0.7 U/mL; Grade 2: 0.7–3.5 U/mL; Grade 3: 3.5–17.5 U/mL; Grade 4: 17.5–50 U/mL; Grade 5: 50–100 U/mL; Grade 6: ≥100 U/mL. Based on the mugwort sIgE grade, patients were categorized into two groups: high reactivity (Grade 4–6) and low reactivity (Grade 1–3). Initial data extracted from the hospital information system comprised 47 candidate features, encompassing demographic information and the sIgE concentration grades of the aforementioned allergens.

Data preprocessing involved the following steps:

(a) **Variable definition:** The binary target variable “Mugwort_IgE_High” was defined as 1 for patients with mugwort sIgE Grade ≥ 4 (high reactivity) and 0 for Grade 1–3 (low reactivity).
(b) **Missing value handling:** Numerical variables with a small amount of missing data were imputed using the median.
(c) **Data standardization:** All continuous predictors were standardized using Z-score normalization prior to modeling.
(d) **Dataset splitting:** The complete dataset was randomly split into a training set and an independent test set in an 8:2 ratio, with a random seed set to 123 to ensure reproducibility.

### Feature selection and machine learning model training

To identify the most parsimonious and predictive feature subset from the 47 candidates, we applied the Least Absolute Shrinkage and Selection Operator (LASSO) regression model on the training set. The optimal regularization parameter *λ* was determined via 10-fold cross-validation using the lambda.1se criterion.

Using the selected features, we constructed and compared four machine learning algorithms: logistic regression (serving as the baseline model), random forest (ntree=200), extreme gradient boosting (XGBoost), and support vector machine (with a radial basis function kernel). All models were trained and their hyperparameters optimized on the training set using the caret package in R, employing 10-fold cross-validation with the area under the receiver operating characteristic curve (AUC) as the optimization metric.

### Model evaluation, interpretation and clinical tool development

Model performance was evaluated on the independent test set. Discrimination was assessed using the AUC, accuracy, sensitivity, specificity, precision, F1-score, Cohen's kappa, Matthews correlation coefficient (MCC), and balanced accuracy. Calibration was evaluated via calibration curves. Model stability was assessed using bootstrap resampling. Clinical utility was examined via decision curve analysis (DCA).

The interpretability of the optimal model was analyzed using the SHapley Additive exPlanations (SHAP) framework. Based on the optimal model, a clinical prediction tool comprising a nomogram and a five-level risk stratification system was developed.

### Model validation

The model's generalizability was assessed on the independent temporal validation set. Additionally, subgroup analyses were performed on the test set based on age (≤18, 19–40, 41–60, >60 years) and sex.

### Statistical analysis

All statistical analyses were performed using R software (version 4.5.0). Continuous variables were assessed for normality using the Shapiro–Wilk test. Based on the distributional characteristics, data are presented as mean ± standard deviation for normally distributed variables or median (interquartile range) for non-normally distributed variables. Categorical variables are presented as frequencies and percentages (n, %).

For group comparisons between low and high mugwort reactivity groups, continuous variables were analyzed using the independent Student's t-test (for normally distributed data) or the Mann–Whitney U test (for non-normally distributed data). Categorical variables were compared using the Chi-square test or Fisher's exact test when expected cell counts were less than 5. All statistical tests were two-sided, and *P*-values < 0.05 were considered statistically significant.

To address the issue of multiple comparisons in the baseline characteristic analysis, the False Discovery Rate (FDR) was controlled using the Benjamini-Hochberg procedure. Variables that remained statistically significant after FDR correction are specifically noted in the results.

## Results

### Baseline characteristics and predictor distribution of the study population

**Figure 2:**
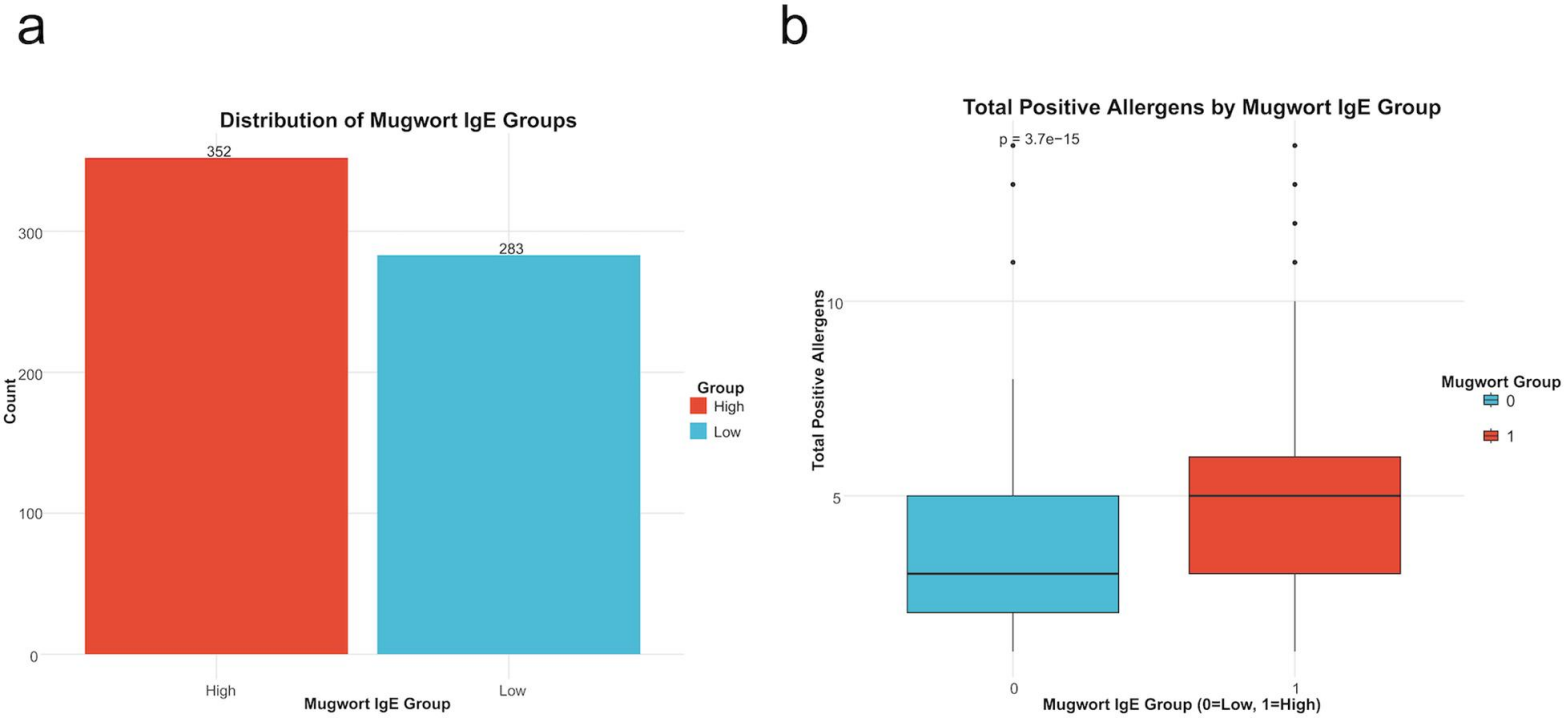
Study population characteristics and distribution of core predictors. **(a)** Bar plot showing the distribution of patients across the primary outcome: mugwort IgE high and low groups. **(b)** Box plots comparing the distribution of the total number of allergen specificities with positive sIgE.

A total of 635 patients were ultimately included in the analysis. The distribution of patients between the mugwort IgE high-reactivity (High) and low-reactivity (Low) groups was largely balanced (Figure 2a). No significant differences were observed between the two groups regarding age (*P* = 0.074) and gender (*P* = 0.215) (Table 1).

**Table 1:** Baseline characteristics of the retrospective study (*N* = 635).

Characteristic	Overall Cohort	Low Mugwort Reactivity (*N* = 283)	High Mugwort Reactivity (*N* = 352)	*P* Value
Demographic Data				
Age, years	35 (23–46)	36 (26–48)	34 (19–45)	0.074
Male sex, *n* (%)	299 (47.1)	144 (44.2)	122 (49.4)	0.215
Allergen Sensitization Profile				
Total number of allergen specificities with positive sIgE	4 (2–5)	3 (2–5)	5 (3–6)	<0.001***
Number of inhalant allergen specificities with positive sIgE	3 (2–5)	3 (2–4)	4 (3–5)	<0.001***
Number of food allergen specificities with positive sIgE	0 (0–1)	0 (0–1)	0 (0–1)	0.186
Has any food allergy, *n* (%)	179 (28.2)	83 (25.4)	75 (30.4)	0.197
Key Inhalant Allergens (Level)				
Mugwort sIgE concentration (Grade 0–6)	4 (3–4)	3 (1–3)	4 (4–4)	<0.001***
Ragweed sIgE concentration (Grade 0–6)	2 (0–3)	0 (0–2)	3 (2–4)	<0.001***
Trees sIgE concentration (Grade 0–6)	1 (0–3)	1 (0–2)	2 (0–3)	<0.001***
Dust mite combination sIgE concentration (Grade 0–6)	0 (0–0)	0 (0–0)	0 (0–1)	<0.001***
Hops level	0 (0–1)	0 (0–0)	0 (0–1)	<0.001***
Key Inhalant Allergens [Positive, *n* (%)]				
Ragweed positive	434 (68.3)	151 (46.3)	213 (86.1)	<0.001***
Trees positive	402 (63.3)	164 (50.2)	183 (73.9)	<0.001***
Dust mite combination positive	145 (22.8)	45 (13.8)	74 (30.1)	<0.001***
Hops positive	182 (28.7)	66 (20.1)	88 (35.5)	<0.001***
Sampling Season, *n* (%)				
Summer	572 (90.1)	–	–	–
Autumn	38 (6.0)	–	–	–
Winter	12 (1.9)	–	–	–
Spring	13 (2.0)	–	–	–

Data are presented as median (interquartile range) for continuous variables and *n* (%) for categorical variables. *P* values were derived from Mann–Whitney U test for continuous variables and Chi-square test for categorical variables.

*** *P* < 0.001 for intergroup comparison.

To further characterize the sensitization profile of the study population, we analyzed the prevalence of sensitization across different allergen categories. Among the 635 patients, sensitization was most common to mugwort (100%, by inclusion criteria) and ragweed (66.3%), followed by tree pollen (62.5%), molds (31.2%), and dust mites (22.4%). A total of 78.9% of patients were sensitized to at least two distinct allergen categories, reflecting a highly polysensitized population. The median number of positive allergen specificities was 4 (IQR: 2–6) overall, with the high-reactivity group having a significantly higher median (5, IQR: 3–7) compared to the low-reactivity group (3, IQR: 2–5) (*P* < 0.001).

The number of allergen specificities with positive sIgE was significantly higher in the high-reactivity group compared to the low-reactivity group (*P* < 0.001; Figure 2b). Among the three allergens that constituted the core predictors of the model—namely ragweed, trees, and dust mites—significant differences in sIgE levels were observed between the two groups. Compared to the low-reactivity group, the high-reactivity group exhibited significantly higher ragweed sIgE concentrations (Ragweed_Level, *P* < 0.001), trees sIgE concentrations (Trees_Level, *P* < 0.001), and dust mite combination sIgE concentrations (Dust_Mite_Combination_Level, *P* < 0.001).

### Feature selection based on LASSO regression

**Figure 3:**
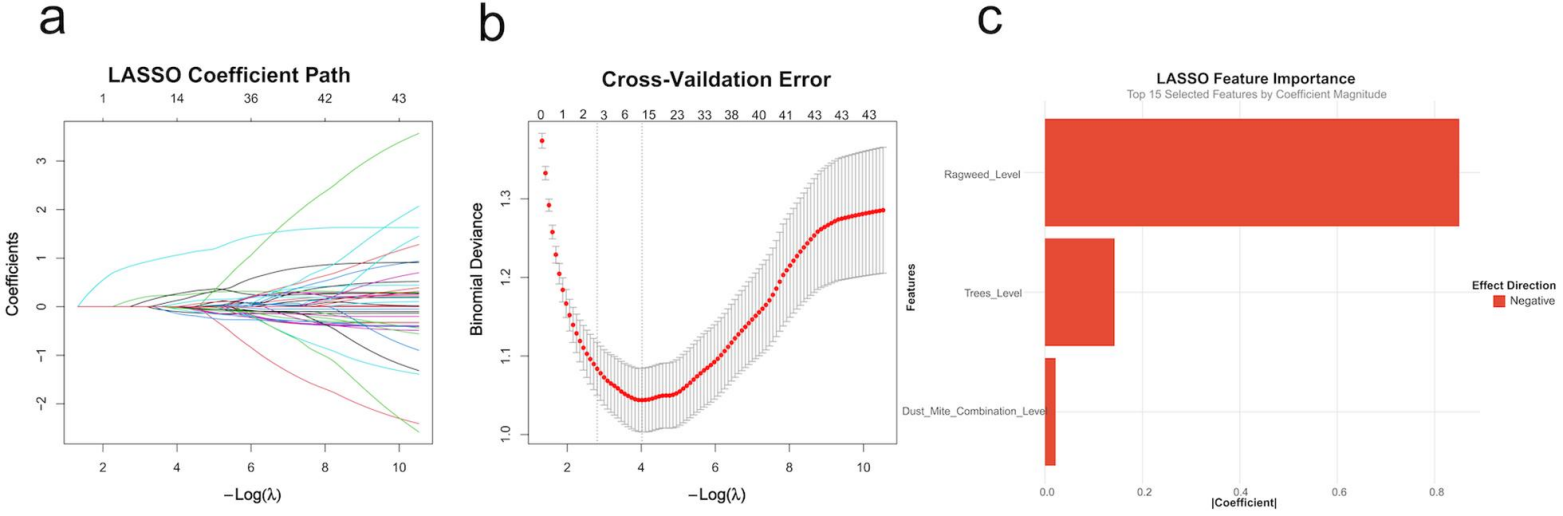
Feature selection using Least Absolute Shrinkage and Selection Operator (LASSO) regression. **(a,b)** Coefficient paths of features against the L1-norm [log(*λ*)]. Each colored line represents the coefficient of a feature. The vertical dashed line indicates the optimal lambda (lambda.1se) chosen by 10-fold cross-validation, which resulted in 3 non-zero coefficients. **(c)** Importance of the three features selected by the LASSO model—ragweed sIgE concentration, trees sIgE concentration, and dust mite combination sIgE concentration—ranked by the absolute value of their standardized coefficients. All selected features negatively contribute to the prediction of high mugwort IgE.

To select the most parsimonious subset of predictive features, we applied the Least Absolute Shrinkage and Selection Operator (LASSO) regression to the 47 candidate features in the training set. As shown in Figures 3a,b, the three selected features, ranked by the absolute value of their coefficients, were ragweed sIgE concentration, trees sIgE concentration, and dust mite combination sIgE concentration (corresponding to the variables Ragweed_Level, Trees_Level, and Dust_Mite_Combination_Level). The LASSO regression coefficients for all selected features were negative (Figure 3c), indicating that—conditional on the other selected features—higher levels of these predictors are associated with a lower probability of being in the high mugwort IgE reactivity group.

It is important to note that the univariate associations shown in Table 1—significantly higher ragweed, tree, and dust mite sIgE levels in the high mugwort IgE group—are not contradictory to the negative coefficients observed in the LASSO multivariate model (Figure 3c). Rather, they illustrate the distinction between marginal and conditional associations. In univariate analysis, these features show positive associations with high mugwort IgE reactivity, likely reflecting that polysensitized individuals generally have higher overall atopic propensity. However, in the multivariate model, after simultaneously accounting for multiple correlated allergen features, the unique conditional contribution of each feature—when holding other features constant—may differ in direction. This phenomenon, where the direction of association reverses after adjusting for other variables, is known in statistics as a suppressor effect and is not uncommon when predictors are correlated. From a biological perspective, this may reflect resource competition within the immune system: among individuals with high overall atopic propensity, those with particularly strong responses to certain allergens (e.g., ragweed, trees) may exhibit a relatively weaker response to mugwort due to competition for limited immune resources, potentially “diluting” or “masking” the mugwort-specific contribution. Thus, the negative coefficients do not negate the positive associations observed in Table 1 but rather reveal the unique conditional contributions of these features in a multivariate context, offering insights into complex immune interactions.

It should be noted that the dust mite feature warrants cautious interpretation. As shown in Table 1, the median dust mite sIgE Grade was 0 in both groups, with 30.1% of patients in the high-reactivity group (vs. 13.8% in the low-reactivity group) showing dust mite sensitization. Although this sensitization rate is lower than that of ragweed and trees, it still represents over 100 patients in the high-reactivity group, providing sufficient variability for model estimation. Notably, LASSO regression inherently performs feature selection; the retention of the dust mite feature in the final model indicates that it provides non-redundant predictive information. If the feature were unstable or lacked genuine predictive value, LASSO would have shrunk its coefficient to zero. Furthermore, the coefficient magnitude of the dust mite feature (Figure 3c) was substantially smaller than those of ragweed and trees, reflecting its relatively minor contribution to the overall model.

Notably, the correlations among these three selected features were weak to moderate (Spearman's |*r*| < 0.45), as shown in Supplementary Figure S1, indicating that they provide relatively independent information and collectively contribute to the model's predictive power.

### Comparison of machine learning model performance

Based on the three core features selected by LASSO, we constructed and comprehensively evaluated four machine learning models (XGBoost, Random Forest, Support Vector Machine, and Logistic Regression) for predicting high mugwort IgE reactivity.

#### (a) Model Discriminatory Ability

**Figure 4:**
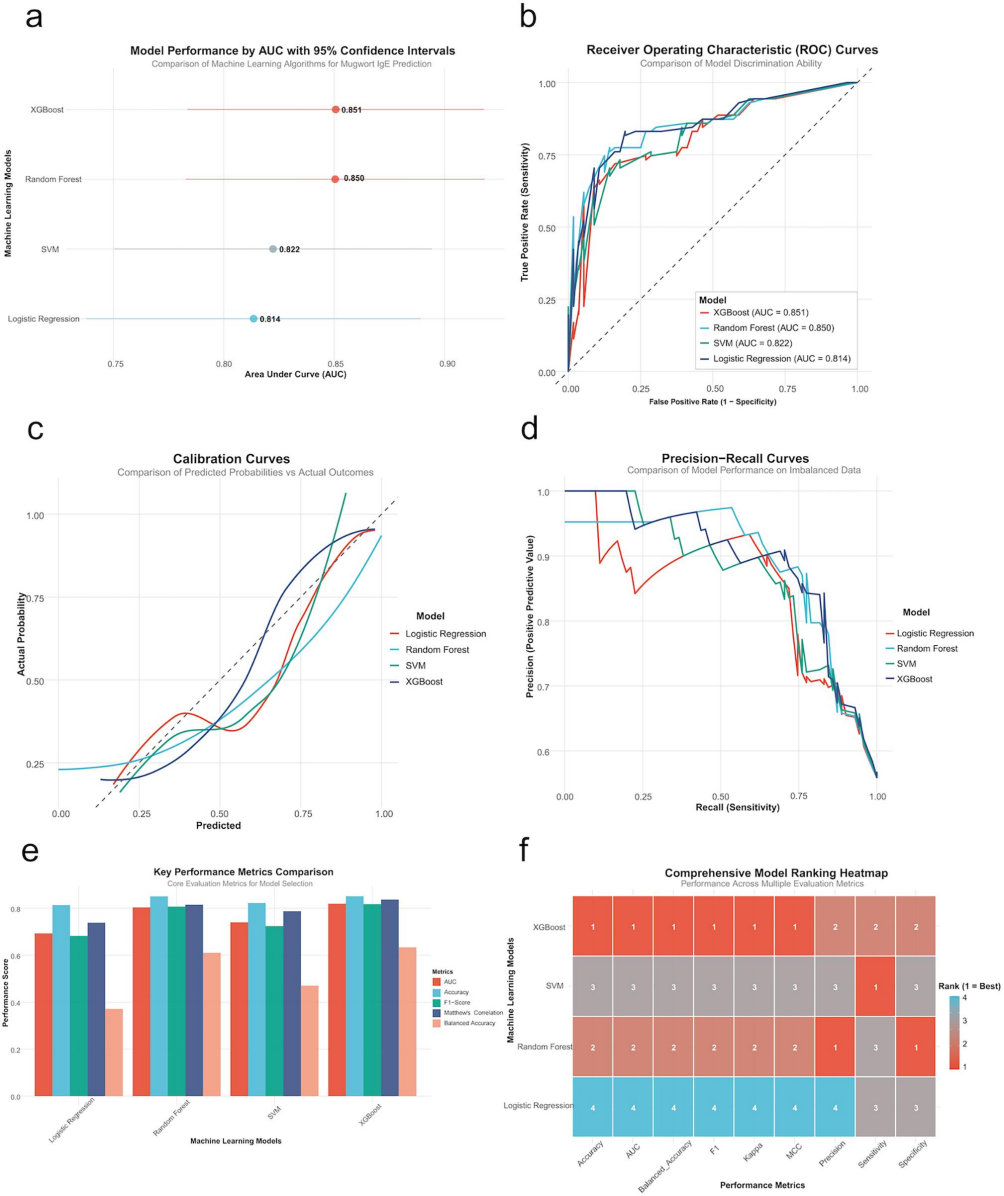
Comparison of the performance of the four machine learning models for predicting mugwort IgE high response. **(a)** Bar plot of the Area Under the Curve (AUC) values with 95% confidence intervals for each model. **(b)** Receiver Operating Characteristic (ROC) curves illustrating the models’ discrimination ability. **(c)** Calibration curves showing the agreement between predicted probabilities and actual outcomes. **(d)** Precision-Recall curves evaluating performance on the dataset. **(e)** Comparison of key performance metrics across the four models. **(f)** Comprehensive model ranking heatmap across multiple evaluation metrics (darker color indicates better rank).

All models demonstrated good discriminatory ability (Figures 4a,b). The XGBoost model achieved the highest AUC of 0.851 (95% CI: 0.781–0.919), performing comparably to the Random Forest model (AUC = 0.850). The Support Vector Machine (AUC = 0.822) and Logistic Regression (AUC = 0.814) models performed slightly less well. The Receiver Operating Characteristic (ROC) curves visually illustrate these results, with the curves for XGBoost and Random Forest nearly overlapping and positioned closest to the top-left corner.

#### (b) Calibration of Predicted Probabilities

The calibration curves (Figure 4c) indicated that the predicted probabilities of the Logistic Regression model aligned most closely with the observed frequencies. The calibration curves for XGBoost and Random Forest lay below the diagonal, suggesting a tendency to overestimate risk probability. The Precision-Recall curve (Figure 4d) showed that the XGBoost model also exhibited the best overall performance, with the largest area under the curve, indicating an optimal balance between accurately identifying positive cases (high precision) and minimizing missed diagnoses (high recall).

#### (c) Comprehensive Performance Evaluation and Ranking

A comprehensive comparison was conducted using five key metrics: AUC, Accuracy, F1-Score, Matthew's Correlation Coefficient (MCC), and Balanced Accuracy (Figure 4e). The XGBoost model ranked first in four of these metrics. A model ranking heatmap (Figure 4f) provides a visual summary: across a total of nine performance and ranking indicators, XGBoost ranked first in seven and second in two, demonstrating the most robust and superior overall performance. Consequently, XGBoost was identified as the optimal model for subsequent analyses.

### Model validation

#### (a) Internal validation: comprehensive assessment on the independent test Set

**Figure 5:**
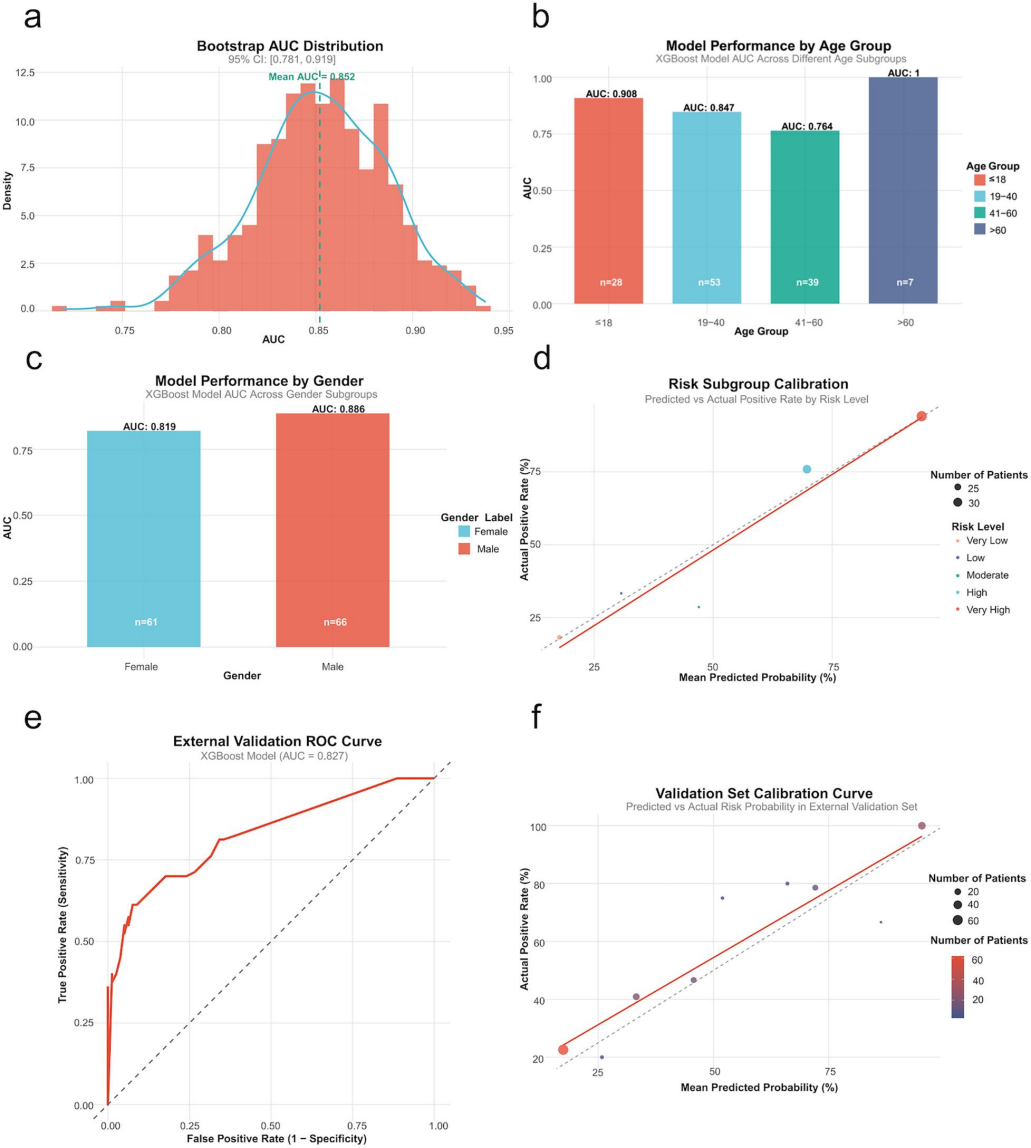
Comprehensive validation of the XGBoost model. **(a)** Distribution of the Area Under the Curve (AUC) from 1,000 bootstrap resamples on the independent test set (*n* = 127), demonstrating the robustness of the model performance estimate. **(b,c)** Subgroup analysis of the XGBoost model's performance (AUC) on the independent test set: **(b)** across different age groups, and **(c)** between genders. **(d)** Calibration plot of predicted probability and actual positive rate for each risk subgroup in the independent test set. **(e)** Receiver Operating Characteristic (ROC) curve of the XGBoost model on the temporal validation set (*n* = 159), achieving an AUC of 0.827. **(f)** Calibration curve for the model's predictions on the temporal validation set, showing the agreement between predicted probabilities and observed outcomes.

Model Stability Assessment via Bootstrap Validation:

To assess the robustness of the optimal XGBoost model's performance, bootstrap resampling (1,000 repetitions) was performed on the independent test set. The results showed a mean bootstrap AUC of 0.852 with a 95% confidence interval of [0.781, 0.919] (Figure 5a), demonstrating stable performance estimation and ruling out overfitting.

Subgroup Analysis of Model Performance:

Subgroup analysis on the independent test set indicated that the XGBoost model maintained good predictive efficacy across different age groups (AUC range: 0.764 to 1.000). The model performed comparably in male (AUC = 0.886) and female (AUC = 0.819) patients, showing no evident bias (Figures 5b,c).

Risk Stratification Performance and Clinical Utility:

Risk stratification calibration demonstrated a high concordance (correlation r = 0.957) between the mean predicted probability for each of the five risk categories and the actual positive rate of patients within those categories in the independent test set (Figure 5d). The distribution of patients, with nearly 50% in combined high and very high-risk categories (Supplementary Figure S2), underscores the clinical necessity of this stratification.

#### (b) External validation: generalizability on the temporal validation Set

To ultimately evaluate the model's generalizability to future patient data, a multi-dimensional validation was performed on an independent temporal validation set (*n* = 159).

The XGBoost model maintained strong discriminatory power in this independent validation set, achieving an AUC of 0.827 (Figure 5e). The calibration curve indicated good overall agreement between the predicted probabilities and the actually observed outcomes (Figure 5f).

### Interpretability analysis of the optimal model

The SHapley Additive exPlanations (SHAP) framework was employed to interpret the optimal XGBoost model. Furthermore, we provided more detailed SHAP dependence scatter plots (Supplementary Figure S3), colored by the patient's true outcome, which further elucidate the model's prediction patterns for positive and negative samples at each feature level.

#### (a) Global Feature Importance

**Figure 6:**
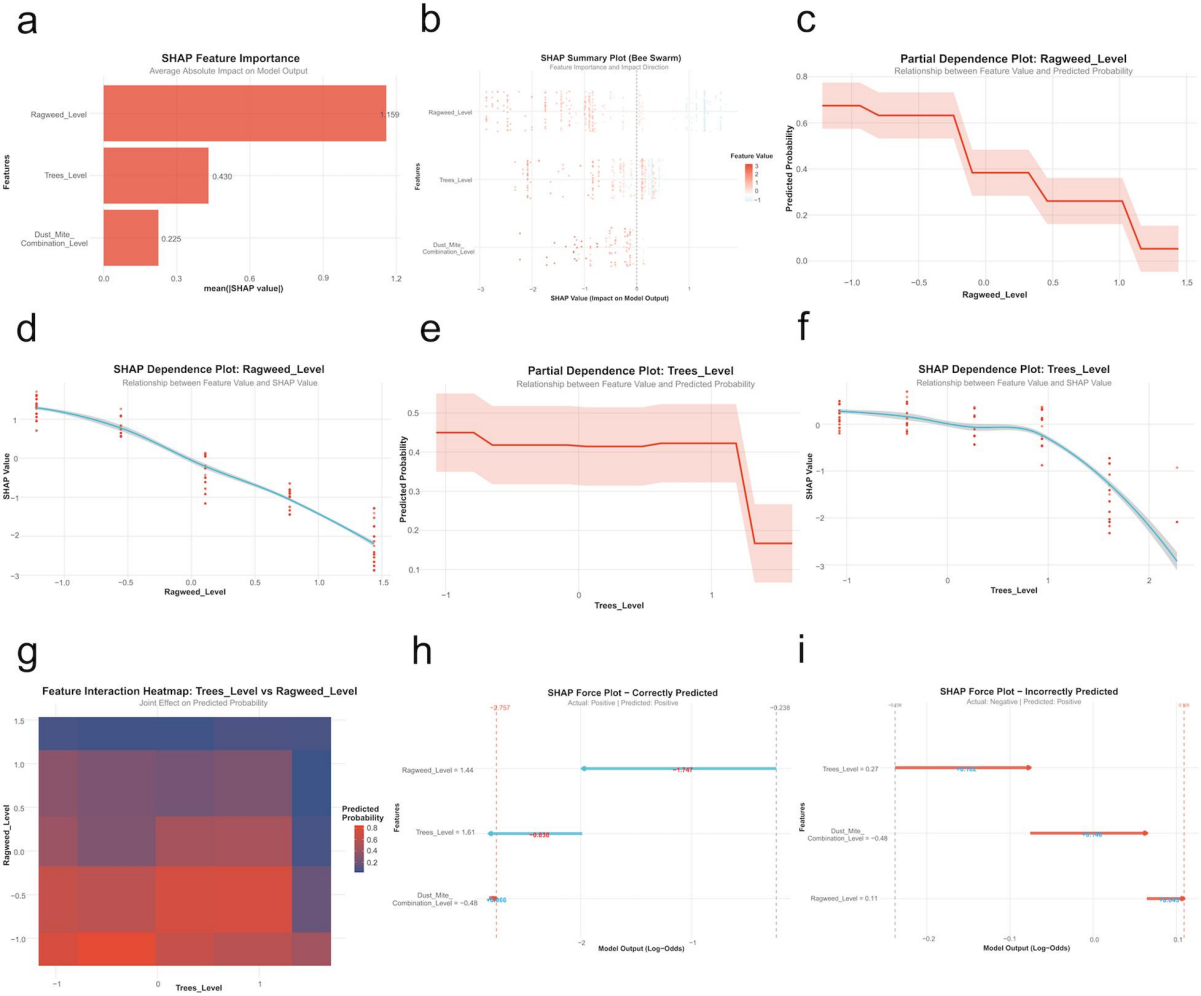
Comprehensive interpretable machine learning analysis of the optimal XGBoost model for mugwort IgE prediction. **(a)** Global feature importance ranking based on mean absolute SHAP values. **(b)** Beeswarm plot summarizing the distribution and direction of feature impacts (red: high feature value, blue: low). **(c,e)** Partial Dependence Plots for Ragweed_Level and Trees_Level, showing the negative relationship between feature values and the average predicted probability. **(d,f)** SHAP dependence plots for the same features, illustrating individual data points and the variation in feature effect. **(g)** Feature interaction heatmap between the two most important features, Trees_Level and Ragweed_Level, colored by the joint predicted probability. **(h,i)** SHAP force plots for a representative **(h)** correctly predicted and **(i)** incorrectly predicted instance, decomposing the model's decision from the base value.

SHAP analysis confirmed that ragweed sIgE concentration (Ragweed_Level) was the strongest **negative** predictor for high mugwort IgE reactivity (mean absolute SHAP value = 1.16), with an influence substantially higher than that of trees sIgE concentration (Trees_Level, 0.43) and dust mite combination sIgE concentration (Dust_Mite_Combination_Level, 0.22) (Figure 6a).

#### (b) Direction and Distribution of Feature Impacts

The SHAP summary beeswarm plot (Figure 6b) revealed a consistent negative impact pattern of the three features on the model output: namely, higher sIgE concentrations (red dots) corresponded to negative SHAP values, leading to a decrease in the predicted risk probability, further confirming their role as negative predictors.

#### (c) Individual Feature Effects and Interactions

Partial Dependence Plots (PDPs) clearly illustrated the global negative correlation between ragweed and trees sIgE concentrations (Ragweed_Level and Trees_Level) and the average predicted probability (Figures 6c,e). SHAP dependence plots revealed variability in feature effects at the individual level (Figures 6d,f), suggesting potential interactions among features. A feature interaction heatmap confirmed the presence of a non-additive joint effect between Ragweed_Level and Trees_Level (Figure 6g).

#### (d) Individual Prediction Explanations

Individual predictions were interpreted using SHAP force plots. In a correctly predicted positive instance (Figure 6h), low Trees_Level and Ragweed_Level values provided positive SHAP contributions, pushing the model output significantly higher from the base value [f(*X*) = −0.238, corresponding to a probability of 44.1%], ultimately resulting in a high-risk prediction. In a negative instance that was incorrectly predicted as positive (Figure 6i), the model still captured positive contributions from its relatively low levels, leading to the misclassification.

### Development and performance of the mugwort IgE prediction nomogram

To translate the optimal predictive model into a clinically practical tool, we developed a visual nomogram based on its core predictors and evaluated its potential clinical application value.

#### (a) Model Coefficients and Feature Weights

**Figure 7:**
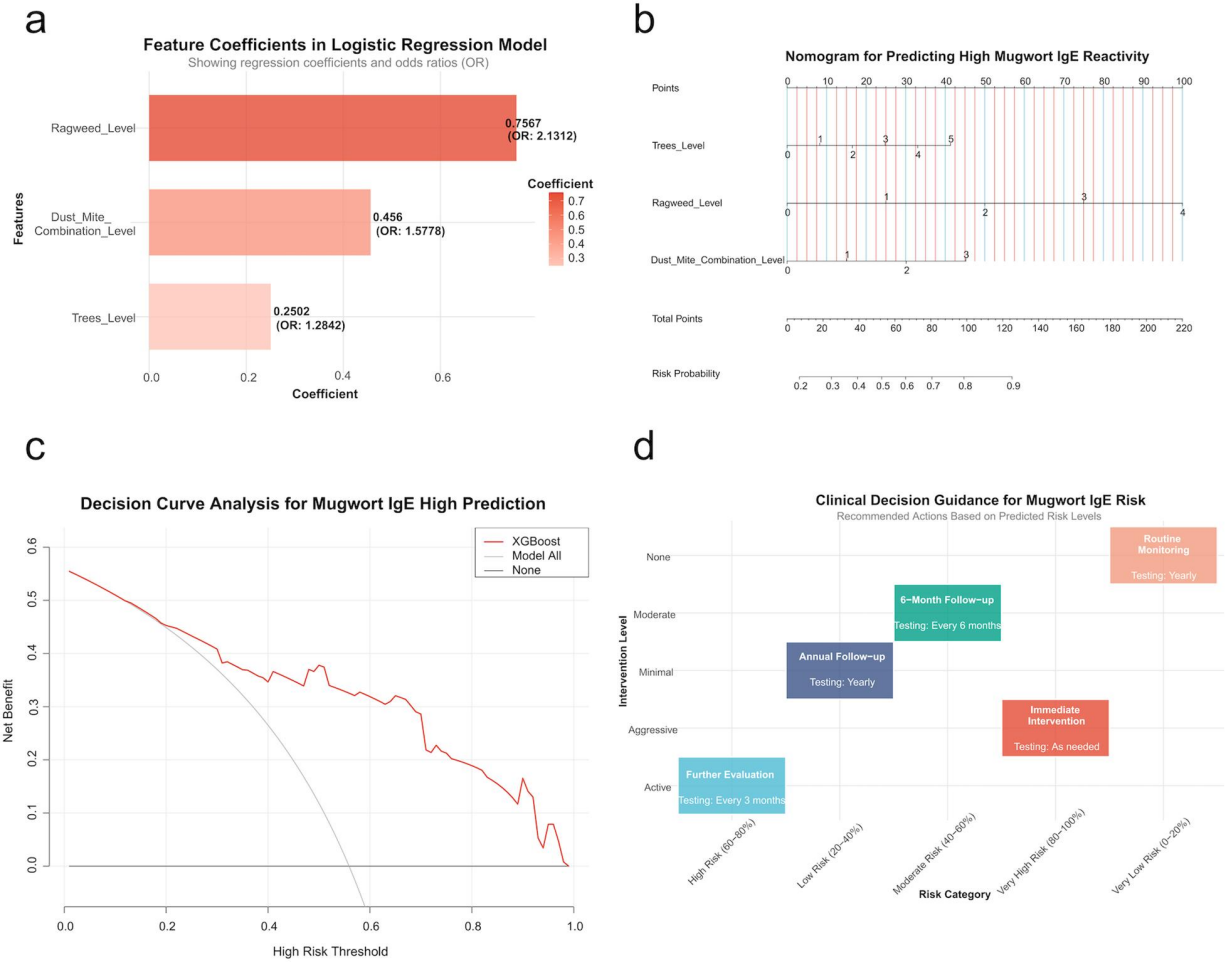
Development and clinical application of the Mugwort IgE prediction nomogram. **(a)** Coefficients and odds ratios of features in the logistic regression model. **(b)** Nomogram for predicting the level of high mugwort IgE reactivity. **(c)** Decision curve analysis of the predictive model based on the training set data. **(d)** Clinical decision and management guidelines based on predicted risk levels.

The weights of each feature for the nomogram were determined within a logistic regression framework (Figure 7a). Ragweed sIgE concentration (Ragweed_Level) exhibited the strongest predictive power (coefficient = 0.757, OR = 2.131), followed by trees sIgE concentration (Trees_Level, coefficient = 0.456, OR = 1.578) and dust mite combination sIgE concentration (Dust_Mite_Combination_Level, coefficient = 0.250, OR = 1.284).

#### (b) Construction of the Clinical Prediction Nomogram

Based on the multivariable analysis results, we constructed a nomogram for the individualized prediction of high mugwort IgE reactivity (Figure 7b). This tool converts the values of each predictor into corresponding points. For Trees_Level (grades 0–5), the point range is 0–22, with the following conversion: Grade 0 → 0 points, Grade 1 → 4 points, Grade 2 → 9 points, Grade 3 → 13 points, Grade 4 → 18 points, Grade 5 → 22 points. For Ragweed_Level (grades 0–5), the point range is 0–68: Grade 0 → 0 points, Grade 1 → 14 points, Grade 2 → 27 points, Grade 3 → 40 points, Grade 4 → 54 points, Grade 5 → 68 points. For Dust_Mite_Combination_Level (grades 0–3), the point range is 0–24: Grade 0 → 0 points, Grade 0.75 → 6 points, Grade 1.5 → 12 points, Grade 2.25 → 18 points, Grade 3 → 24 points. The total points (range 0–140) are calculated by summing the points from the three predictors, and the corresponding individualized risk probability (16.9%–94.5%) can be read from the bottom axis.

#### (c) Clinical Decision Curve Analysis

Decision Curve Analysis (DCA) showed (Figure 7c) that across a wide range of decision threshold probabilities (0.1–0.8), the use of the XGBoost predictive model (the “XGBoost Model” curve) for clinical decision-making yielded a higher net benefit than the extreme strategies of “Treat All” or “Treat None”. The model achieved the maximum net benefit (0.5546) at a threshold probability of 0.01 and maintained superior performance in the medium-to-high threshold interval (0.3–0.7).

#### (d) Risk Stratification and Clinical Application

Based on the predicted probabilities, patients were stratified into five risk categories (Very Low: 0%–20%; Low: 20%–40%; Intermediate: 40%–60%; High: 60%–80%; Very High: 80%–100%), and corresponding management pathways were established (Figure 7d). In the independent test set, the actual positive rate was 85% in the high-risk group (>60%), compared to 25.8% in the low-risk group (<40%).

The nomogram and risk stratification system provide clinicians with intuitive, individualized risk assessment results. As shown in Supplementary Figure S4, management strategies can be tailored based on the patient's specific risk category (e.g., “Intermediate risk” 47.3%, “Very High risk” 97.4%).

## Discussion

It should be noted that the immunological mechanisms proposed in this study (including the “molecular masking” and “immunodominance” hypotheses) are speculative interpretations based on observational data. They are intended to generate hypotheses for future mechanistic studies rather than to establish definitive causal relationships.

In this study, we identified three core predictors—specific IgE concentration to ragweed, trees, and dust mites—from 47 inhalant allergen features using LASSO regression, and subsequently developed a high-performance XGBoost machine learning model to predict high mugwort sIgE reactivity. The model demonstrated robust discriminatory power (AUC: 0.827) in the temporal validation set, through the SHAP framework, revealed a seemingly counterintuitive immunological phenomenon: after adjusting for other allergen sensitizations, sensitization to ragweed, trees, and dust mites served as strong negative predictors for high mugwort IgE reactivity. This finding is termed “counterintuitive” because clinically, polysensitized patients are generally expected to exhibit stronger responses to major allergens, not weaker; indeed, univariate analysis showed significantly higher sIgE levels for these allergens in the high-reactivity group. However, in the multivariate model, after accounting for inter-allergen interactions, the unique conditional contribution of these features reversed direction, suggesting complex resource competition within the immune system. Ultimately, we successfully translated these findings into a comprehensive decision-support tool comprising a nomogram, risk stratification, and clinical management pathways.

### Immunological mechanism: From cross-reactivity to immunodominance

The immunological mechanisms proposed below should be considered speculative hypotheses based on observational data, intended to generate directions for future mechanistic studies rather than to establish definitive causal relationships. It is important to clarify that “negative predictors” in this study refer to the conditional associations in the multivariate model: when other allergen sensitization levels are held constant, higher levels of these features are associated with a lower probability of high mugwort IgE reactivity. This does not imply that these features are negatively correlated with mugwort IgE levels in univariate analysis—in fact, univariate analysis showed positive associations (Table 1). The discrepancy between conditional negative and marginal positive associations lies at the heart of our findings and justifies the use of the term “counterintuitive.” The most striking finding of this study is the consistent negative predictive effect of three key inhalant allergen features on mugwort IgE reactivity. This consistent negative correlation provided the crucial biological foundation for building our machine learning model. A complete chain of evidence, from baseline comparisons and LASSO regression to SHAP analysis, collectively confirms that these allergen sIgE levels are robust negative predictors of high mugwort sIgE reactivity.

#### (a) The “Molecular Masking” Hypothesis and the Dual Role of Cross-Reactivity

It is first necessary to distinguish between two related but distinct immunological concepts. Cross-reactivity refers to the ability of IgE antibodies to bind to structurally similar molecules from different allergen sources, such as profilins found in mugwort (Art v 4), birch (Bet v 2), and timothy grass (Phl p 12) (5, 10). Co-sensitization, in contrast, refers to independent IgE responses to multiple unrelated major allergens, such as concurrent sensitization to the mugwort major allergen Art v 1 and the ragweed major allergen Amb a 1 (11, 12). In the present study, because we used crude extract testing rather than component-resolved diagnostics, we cannot distinguish whether patients' IgE responses are directed against major allergens or cross-reactive pan-allergens (5, 11). Therefore, the observed conditional negative association between ragweed, tree, and dust mite sIgE levels and high mugwort IgE reactivity may reflect complex immune interactions in the context of co-sensitization (11–13). We propose the “molecular masking” hypothesis as a potential explanation: if high sIgE levels to ragweed, trees, and dust mites reflect, at least in part, strong responses to cross-reactive pan-allergens (e.g., profilins and CCDs) (5, 14), these low-affinity but broad-spectrum IgE antibodies (10)may “mask” or dilute the contribution of mugwort-specific IgE within the total IgE repertoire. This could manifest statistically as a negative conditional contribution after adjusting for overall polysensitization. Component-resolved diagnostics are needed to directly test this hypothesis (5, 11).

#### (b) Theoretical Support from “Immunodominance” and Antigen Competition

Our findings align closely with the immunological concept of “immunodominance”: when multiple potential antigenic epitopes compete, the immune system preferentially mounts a strong response to the immunodominant epitope(s) (15). Zbîrcea et al. found that strong sensitization to the major ragweed allergen Amb a 1 was the dominant factor in ragweed pollen allergy (16). This phenomenon is recognized and applied in standardized allergen extracts (17). We speculate that in some patients, a strongly immunodominant response to allergen from ragweed, trees, or dust mites “wins” the immune competition, thereby partially “suppressing” the immune response against mugwort, manifesting as a negative predictive relationship. This mechanism might also explain the reported relatively lower sensitivity of ImmunoCAP for seasonal allergens in patients sensitized to house dust mites (HDM) (18),providing corroborative evidence from clinical diagnostics for immunodominance-induced response bias (19).

Certainly, cross-reactivity is more frequently reported to be associated with co-sensitization and exacerbation of symptoms (12). This seemingly paradoxical phenomenon may stem from differences in patient populations, geographic allergen distribution, and the specific allergen molecules involved (13). For instance, Wu et al., using latent class analysis (LCA), identified a patient subclass (Class 1) characterized by “profilin co-sensitization,” which indeed showed higher skin prick test positivity rates to multiple pollens (11). This creates an interesting tension with our “suppression” hypothesis. A plausible explanation is that cross-reactivity may determine the “breadth” of sensitization (20), while immunodominance determines the “intensity” of the response. This underscores the need for future studies to incorporate component-resolved diagnosis (CRD) for more refined stratified analyses (21, 22). Indeed, the LCA study by Wu et al., which clearly identified distinct sensitization phenotypes (11), provides important clinical corroboration for understanding the net population-level effect observed in our study.

It should be noted that the dust mite feature should be interpreted with caution in this study. Although retained in the LASSO model, this feature had a relatively low sensitization rate in our cohort (22.4% overall, 30.1% in the high-reactivity group), and its biological and predictive relevance requires further validation in populations with higher dust mite sensitization rates.

In addition to the “molecular masking” and “immunodominance” hypotheses proposed above, alternative explanations for the observed conditional negative associations should be considered. First, different stages of the allergic march may play a role in polysensitized individuals (4). In our study population, the high mugwort IgE reactivity group may represent a later stage of the allergic march, where responses to “earlier” allergens such as ragweed or trees may have plateaued or are being outcompeted by other responses. Second, individual differences in clinical reactivity thresholds may influence our group classification based on sIgE grades. Some patients may exhibit clinical reactivity to mugwort at lower sIgE levels, while others may remain asymptomatic even at higher levels, potentially affecting the assignment to “high” vs. “low” reactivity groups. Third, timing and duration of allergen exposure vary across geographic regions and individuals, which may shape sensitization patterns (2). In the Ningxia region, the pollen seasons for ragweed and mugwort overlap but have distinct peaks, and the order and intensity of exposure may influence the outcomes of immune competition. Finally, the semi-quantitative nature of our detection method (grades 0–6) may not fully capture subtle variations in sIgE concentrations, potentially affecting the ability of multivariate models to resolve complex nonlinear relationships. These alternative explanations do not preclude the possibility of “molecular masking” or “immunodominance” mechanisms but emphasize the need for more refined component-resolved diagnostics and longitudinal studies to elucidate the underlying processes.

### Model value and clinical translation

Recently, multiplex-specific immunoglobulin E (IgE) platforms have emerged for better selection of patients at risk for anaphylaxis and have improved the selection criteria for patients undergoing allergen immunotherapy, including novel regimes such as oral immunotherapy (23, 24). Within this context, our study provides a novel approach to characterizing the complex relationships between sensitization to different allergen sources. Importantly, we do not propose the model as a clinical prediction tool to replace direct mugwort IgE measurement; rather, we emphasize its value in revealing patterns of co-sensitization that may inform our understanding of how immune responses to different allergens interact. These findings may ultimately contribute to more refined stratification of allergic patients and generate testable hypotheses for future mechanistic studies.

#### (a) Unique Advantage of Machine Learning in Capturing Complex Immune Interactions

The XGBoost model constructed in this study demonstrated excellent performance in distinguishing high mugwort sIgE reactivity. Its AUC on the temporal validation set (0.827) was highly comparable to that on the independent test set (0.851), demonstrating the model's good generalizability. This highlights the powerful capability of machine learning algorithms to capture complex nonlinear relationships and interactions among allergens, as revealed by the SHAP analysis. Such complexity is challenging for traditional statistics-based models relying on linear assumptions to fully capture (11).

#### (b) Interpretability Paves the Way for Clinical Trust

Although high-accuracy predictive models hold great potential, their “black-box” nature hinders clinical adoption. This study employed the SHAP framework to deconstruct the optimal model, which not only confirmed ragweed-specific IgE level (Ragweed_Level) as the strongest predictor but, more importantly, enabled the interpretation of individual predictions. This approach aligns with the rationale of cutting-edge studies like Multimodal-AlgPro, which facilitate epitope discovery by identifying interpretable sequence motifs (25). Clinicians can clearly trace how elevated ragweed or tree IgE concentrations contribute to an increased predicted probability of mugwort allergy risk in a patient. This ability to translate complex models into intuitive clinical insights is key to building clinical trust.

#### (c) Implementation of the Decision-Support Tool and Validation of Clinical Utility

The nomogram we developed translates the complex model into a visual tool, and the decision curve analysis demonstrated that its use provides a higher net clinical benefit across a wide range of decision thresholds compared to the “treat-all” or “treat-none” strategies. Based on the predicted probabilities, we stratified patients into five risk tiers with corresponding management suggestions as a reference: very low risk (0%–20%): routine annual monitoring; low risk (20%–40%): annual follow-up and basic screening; intermediate risk (40%–60%): follow-up every 6 months with comprehensive allergen testing; high risk (60%–80%): assessment every 3 months and consideration of specialist referral; very high risk (>80%): immediate clinical intervention and individualized intensive therapy. It should be emphasized that these risk stratifications and management suggestions should be considered an exploratory framework rather than established clinical guidelines, and their clinical application requires further validation in prospective studies. Data from the independent test set indicated good discriminatory ability of this stratification system, with an actual positive rate of 85% in the high-risk group (>60%), compared to only 25.8% in the low-risk group (<40%). Subgroup analysis further indicated that the model maintained robust performance across patients of different ages and genders.

This stratification system aids in optimizing healthcare resource allocation and enables precise allergy risk management. It aligns well with the recently advocated biomarker-based risk stratification management concept in allergic diseases (26). A similar approach was successfully applied in a large-scale predictive model for childhood asthma diagnosis (27), further supporting the feasibility and considerable potential of combining machine learning predictive models with DCA to optimize the clinical management of allergic diseases.

## Limitations

This study has several limitations. First, as a single-center retrospective study, the generalizability of our findings may be limited, and future multi-center prospective studies are warranted for validation. Second, our analysis was based on sIgE levels to crude allergen extracts without component-resolved diagnosis, thus we could not precisely elucidate the specific mechanisms underlying the observed “immunodominance” suppression phenomenon at the molecular level. Potential residual confounding factors, such as unmeasured genetic or environmental exposures, might also influence the results. Finally, although the model performed well on the temporal validation set, its generalizability to broader geographical populations and different clinical settings requires further evaluation. Additionally, the dust mite feature should be interpreted with caution, as the majority of patients in this cohort were not sensitized to dust mites (median grade 0 in both groups). Although retained in the LASSO model, its contribution was relatively minor, and its biological and predictive relevance requires further validation in populations with higher dust mite sensitization rates.

## Conclusion

In conclusion, from a novel immunodominance perspective, this study discovered and characterized a pattern where sensitization to ragweed, trees, and dust mites shows conditional negative associations with high mugwort IgE reactivity after adjusting for other allergens. Using an interpretable machine learning approach, we identified and modeled these complex co-sensitization patterns based on routine multi-sIgE data. This work proposes a new mechanistic hypothesis regarding immune interactions in pollen-sensitized patients and provides a framework for characterizing co-sensitization patterns that may inform future mechanistic and longitudinal studies.

## Data Availability

The datasets used and/or analysed during the current study are available from the corresponding author on reasonable request.

## Supplementary material

Supplementary Figure S1

Correlation heatmap among the three core predictive features.

Supplementary Figure S2

Distribution of patients across the five clinical risk categories in the independent test set.

Supplementary Figure S3

Detailed SHAP dependence plots for (a) Trees_Level, (b) Ragweed_Level, and (c) Dust_Mite_Combination_Level.

Supplementary Figure S4

Examples of indiv idualized risk predictions for representative patients from each risk category.
