# Supplementary figures and images for: An immunodominance perspective on a paradoxical phenomenon: discovery and modeling of ragweed and tree sensitization as negative predictors for high mugwort IgE reactivity
Source: Front Allergy. 2026 Jun 9;7:1758315. doi: 10.3389/falgy.2026.1758315 (PMC13287044; doi:10.3389/falgy.2026.1758315)

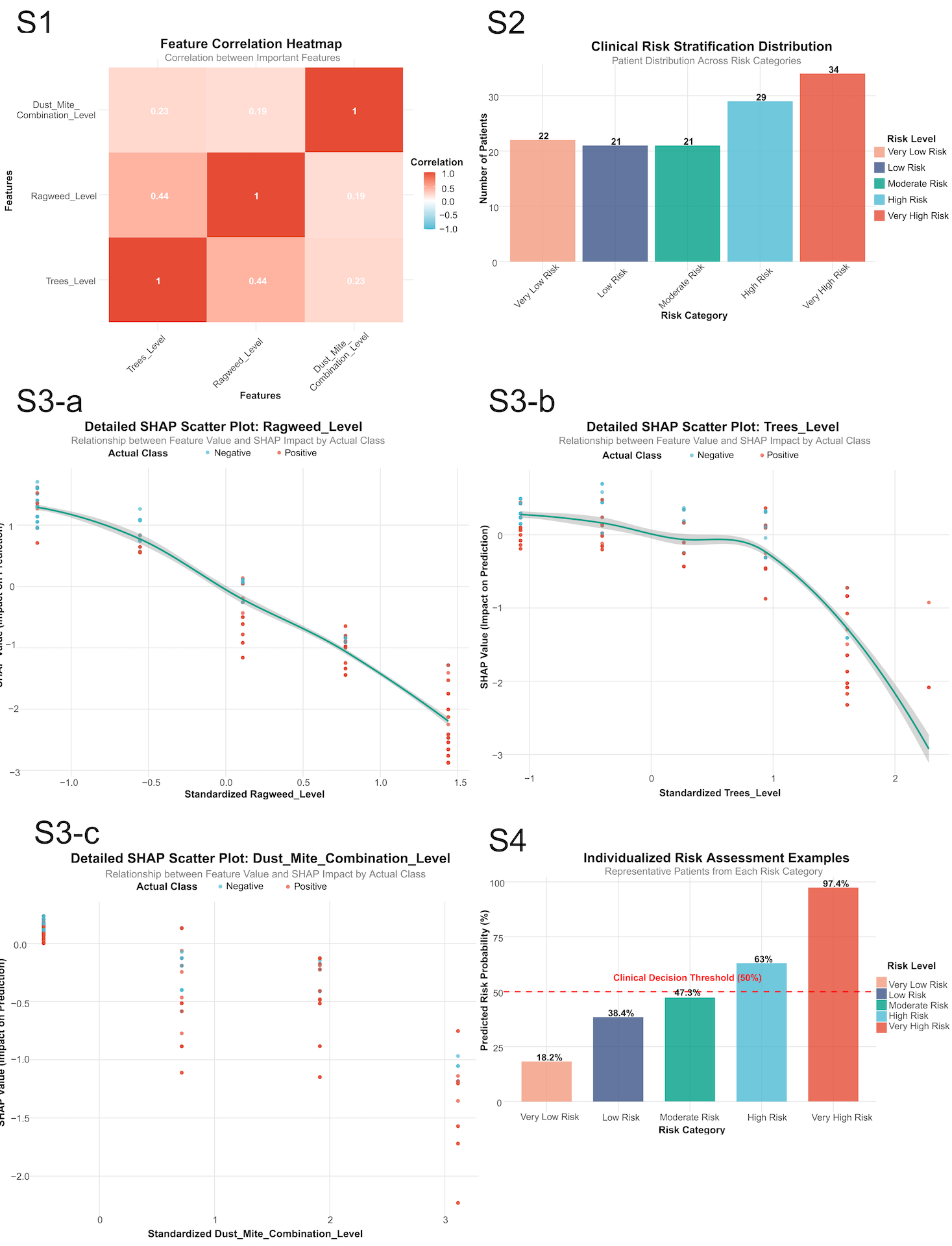

Supplement: Supplementary file 1 [file Image1.tif]
